# (Non)Parallel developmental mechanisms in vertebrate appendage reduction and loss

**DOI:** 10.1002/ece3.8226

**Published:** 2021-10-22

**Authors:** Samantha Swank, Thomas J. Sanger, Yoel E. Stuart

**Affiliations:** ^1^ Department of Biology Loyola University Chicago Chicago Illinois USA

**Keywords:** convergence, evolutionary development, gene regulatory networks, Hedgehog, Hox genes, limb loss, Pitx1

## Abstract

Appendages have been reduced or lost hundreds of times during vertebrate evolution. This phenotypic convergence may be underlain by shared or different molecular mechanisms in distantly related vertebrate clades. To investigate, we reviewed the developmental and evolutionary literature of appendage reduction and loss in more than a dozen vertebrate genera from fish to mammals. We found that appendage reduction and loss was nearly always driven by modified gene expression as opposed to changes in coding sequences. Moreover, expression of the same genes was repeatedly modified across vertebrate taxa. However, the specific mechanisms by which expression was modified were rarely shared. The multiple routes to appendage reduction and loss suggest that adaptive loss of function phenotypes might arise routinely through changes in expression of key developmental genes.

## Graphical abstract

Appendages have been independently reduced or lost hundreds of times during vertebrate evolution. Despite that selection seems to routinely favor a reduced appendage, we found that the specific mechanisms to appendage reduction and loss are rarely shared, even when the same genes were involved.

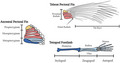

## 1 INTRODUCTION

The vertebrate appendage demonstrates substantial diversity in form and function, having evolved into fins, wings, flippers, claws, hooves, and myriad other structures. Appendage reduction and loss is also a significant component of vertebrate appendage evolution. Repeated, independent instances of appendage reduction and loss offer an opportunity to investigate the extent to which the developmental bases of phenotypic evolution are shared and unique (i.e., (non)parallel) across vertebrate lineages (Bolnick et al., 2018).

Here, we review molecular pathways involved in appendage development to ask whether shared or unique genetic and developmental mechanisms are involved in independent instances of vertebrate appendage reduction and loss. For consistency, we chose to use the nomenclature rules usually reserved for mouse and rat (*Gene* and PROTEIN) throughout our review. Because there are no established guidelines for the discussion of regulatory elements, enhancer symbols will be capitalized and italicized (*ENHANCER*) (Table 1).

**TABLE 1:** Gene and enhancer abbreviations

Gene symbol	Gene name
*Shh*	Sonic Hedgehog
*Gli3*	GLI Family Zinc Finger 3
*Hox*	a gene family comprising a subset of homeobox genes
*Wnt8c*	Wingless‐related integration site 8c
*Wnt2b*	Wingless‐related integration site 2b
*Tbx5*	T‐box transcription factor 5
*Tbx4*	T‐box transcription factor 4
*Pitx1*	Pituitary homeobox transcription factor 1
*Fgf8*	Fibroblast growth factor 8
*Hand2*	Heart and neural crest derivatives expressed 2
*Ptch1*	Protein Patched homolog 1
*Gli1*	GLI Family Zinc Finger 1
*Grem1*	Gremlin1
*Msx2*	Msh homeobox 2
*Bmp4*	Bone morphogenetic protein 4
*Nkx2.5*	Nkx2 homeobox 5
*Cux1*	Cut‐like homeobox 1
*Ihh*	Indian Hedgehog
*Pthrp*	Parathyroid related protein
*C2cd3*	C2 Calcium Dependent Domain Containing 3

Metabolite symbol	Metabolite name
RA	Retinoic Acid

Enhancer symbol	Enhancer name
*PELA*	Pelvic enhancer A
*PELB*	Pelvic enhancer B
*ZRS*	Z(one of polarizing activity) Regulatory Sequence
*GCR*	Global Control Region

Comparing the molecular drivers of appendage reduction and loss across vertebrate clades required that we find taxa that (a) show appendage loss or reduction and (b) have data on the molecular and developmental components driving reduction. Though there are hundreds of independent instances of lost or reduced appendage elements reported for vertebrates, we found only a handful of taxa for which the molecular pathways involved are described even in part, likely limited by the difficulty of studying development in nonmodel organisms.

The cases we did find span 450 million years of vertebrate evolution, from teleost fish to mammals (López et al., 2016). To address generality in appendage loss and reduction across vertebrates, we therefore must discuss homology between teleost fins and tetrapod limbs.

**FIGURE 1:**
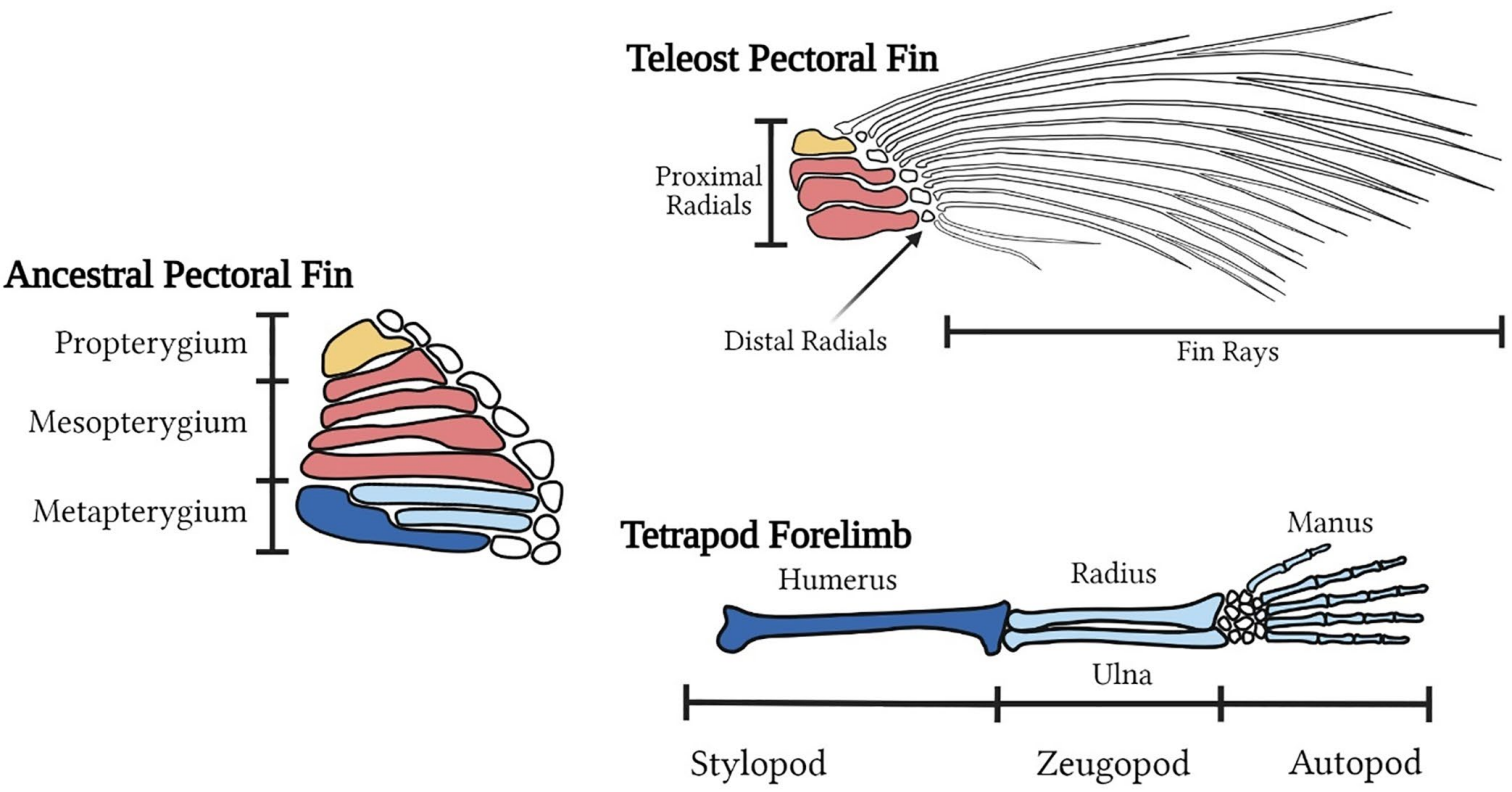
The teleost pectoral fin is based on zebrafish fin morphology, while the tetrapod forelimb is based on human anatomy. Elements of the ancestral pectoral fin are retained and modified in extant vertebrates: Appendage structures are colored to reflect their evolutionary origins. The propterygium (yellow) and mesopterygium (red) were retained and modified in teleost evolution while the metapterygium (dark and light blues) makes up the tetrapod limb. The proximal portion of the metapterygium (dark blue) likely forms the stylopod, while the more distal elements (light blue) were likely elaborated into the distal limb structures (Ahn & Ho, 2008; Don et al., 2013; Freitas et al., 2007; Hawkins et al., 2021)

Teleost fins and tetrapod limbs arose by modifications to the paired fins of their last common ancestor and are superficially similar in position and function (Hall, 2007). Ancestral gnathostome fins were composed of long‐bone segments arranged into three structures along the anteroposterior axis: the propterygium, the mesopterygium, and the metapterygium (Coates, 1994; Don et al., 2013; Hawkins et al., 2021) (Figure 1). In teleosts, the propterygium and mesopterygium form the fins, whereas the metapterygium is lost (Coates, 1994; Don et al., 2013; Hawkins et al., 2021) (Figure 1). In contrast, only a modified metapterygium is retained in tetrapod limbs (Coates, 1994; Don et al., 2013; Hawkins et al., 2021). Thus, the teleost fin and the tetrapod limb are derived from distinct tissues.

However, despite originating from different tissues, a sort of “deep homology” underlies fin and limb development (Shubin et al., 1997, 2009). That is, much of the genetic architecture controlling appendage development is shared between teleosts and tetrapods (Hall, 2007). For example, the Hedgehog pathway plays a role in anteroposterior appendage patterning and maintaining downstream gene expression in both fish and tetrapods (Chiang et al., 2001; Lettice et al., 2003; Ros et al., 2003; Sagai et al., 2005). Alterations to this signaling pathway result in aberrant appendage development and morphology in both clades: experimental loss of *Shh* expression resulted in truncated limbs in mice and in fin absence in the teleost medaka (*Oryzias latipes*) (Chiang et al., 1996; Letelier et al., 2018; Sagai et al., 2005). Similarly, the expression and function of *Gli3*, a *Shh* antagonist, is conserved from fish to tetrapods (Letelier et al., 2020). *Gli3*‐knockout medaka grow extra fin elements; *Gli3*‐deficient mice develop a similar polydactyl phenotype (Letelier et al., 2020; Litingtung et al., 2002; Lopez‐Rios et al., 2012; te Welscher, Zuniga, et al., 2002).

Regulation of Hox genes, a gene family important for embryo patterning in most animals, is also shared in teleost fins and tetrapod limbs (Ahn & Ho, 2008; Cohn & Tickle, 1999; DuBuc et al., 2018; Hall, 2007; Parrish et al., 2009; Ramos et al., 2012; Ryan et al., 2007; Scott, 1993; Tanaka et al., 2005). For example, Hox genes are expressed in three phases in the pectoral appendage of zebrafish and chick; orthologous genes are expressed in similar regions of the appendage during each phase (Ahn & Ho, 2008).

**FIGURE 2:**
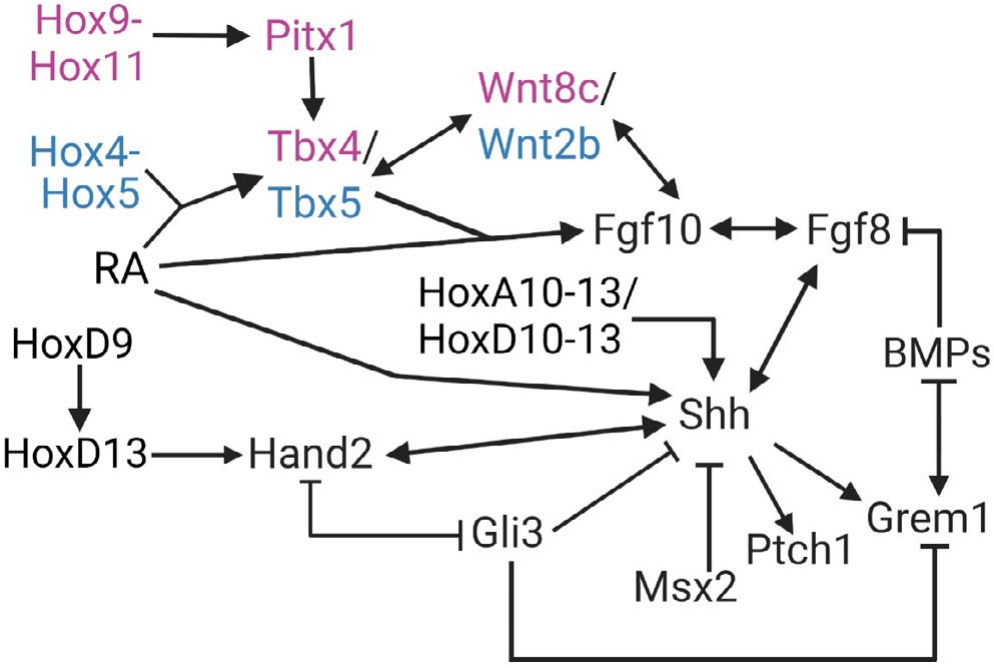
A simplified gene regulatory network implicated in vertebrate appendage development. Genes symbols coded in magenta are unique to the hindlimb, while those in blue are unique to the forelimb (Butterfield et al., 2009; Charité et al., 2000; Delgado et al., 2021; Delgado & Torres, 2015; Fernandez‐Teran et al., 2000; Hockman et al., 2008; Jin et al., 2019; Lafage‐Proust, 2015; McQueen & Towers, 2020; Minguillon et al., 2012; Ng et al., 2002; Nishimoto et al., 2015; Tanaka et al., 2005; te Welscher, Fernandez‐Teran, et al., 2002; Xu & Wellik, 2011; Zúñiga, 2015)

For further examples, orthologs of *Tbx5* and *Tbx4* are required for formation of anterior and posterior appendage, respectively (Bickley & Logan, 2014; Garrity et al., 2002; Minguillon et al., 2005; Naiche & Papaioannou, 2003, 2007; Takeuchi et al., 2003). *Pitx1* expression is similar in the developing posterior appendage of teleosts and tetrapods and induces *Tbx4* expression in both clades as well (Figure 2) (Cole et al., 2003; Duboc & Logan, 2011; Infante et al., 2013; Logan & Tabin, 1999; Marcil et al., 2003; Tickle & Cole, 2004). Altogether, we suggest that there is sufficient homology between fins and limbs to assess (non)parallelism in the genetic basis of appendage loss and reduction across the vertebrate phylogeny.

Having supported homology between fins and limbs, we now define appendage reduction and loss, the main criteria for taxon inclusion for this review. Defining “loss” is straightforward: the absence of one or more bones from the appendage, from pelvic or pectoral girdles to fin rays or digits. “Reduction” has had a more varied definition over its study (Bickley & Logan, 2014; Brandley et al., 2008; Chiang et al., 2001; Greer, 1991; Klepaker et al., 2013; Kragesteen et al., 2018; Thompson et al., 2018; Wiens et al., 2006). For our review, we consider “reduction” to be a diminishment in the relative length or width of at least one bone in the appendage.

We now divide the rest of our review by clade, appendage, and modification type to allow for comparisons between taxa and establish if the same molecular mechanisms are used for appendage reduction or loss by distantly related vertebrates. While the complex gene regulatory networks dictating appendage development may offer numerous routes to reduction and loss, we found that these phenotypes most often resulted from modified regulation of the same of key developmental genes (Table 2).

**TABLE 2:** Summary of molecular mechanisms of reduction and loss

Genus	Reduction/Loss type	Molecular modification^a^
*Gasterosteus*	Pelvic fin and girdle reduction/Loss	*Pitx1* (*PelA*, *PelB*), *Tbx4* ^b^ Reduced or missing initiation signaling
*Pungitius*	Pelvic fin and girdle reduction/Loss	*Pitx1* Reduced or missing initiation signaling
*Takifugu*	Pelvic fin and girdle loss	*HoxD9a* Missing positional signaling
*Python*	Hindlimb and pelvic girdle reduction & loss	*Shh* (*ZRS*), *Fgf8* ^b^ Attenuated outgrowth signaling
*Hemiergis*	Digit loss	*Shh* Reduced signal duration
*Stenella*	Hindlimb loss and pelvic girdle reduction	*Hand2*, *Shh* ^b^, *Fgf8* ^b^ Missing outgrowth signaling
*Trichechus*	Hindlimb loss and pelvic girdle reduction	*Pitx1* ^†^ Missing initiation signaling
*Sus*	Digit reduction and loss	*Ptch1*, *Gli1* ^b^, *HoxD* ^b^, *Grem1* ^b^, *Fgf8* ^b^ Reduced outgrowth signaling
*Bos*	Digit loss	*Ptch1* (LRM), *Gli1* ^b^, *HoxD* ^b^, *Grem1* ^b^, *Fgf8* ^b^ Missing outgrowth signaling
*Camelus*	Digit loss	*Msx2*, *Bmp4* Apoptosis
*Equus*	Digit loss	*Msx2, Bmp4* Apoptosis
*Dipus*	Digit loss	*Msx2, Bmp4* Apoptosis
*Carollia*	Forelimb reduction	*HoxD13*, HoxD genes Differential growth rate
*Myotis*	Forelimb reduction	*HoxD13*, HoxD genes (*GCR*, *BAR116*) Differential growth rate
*Rhinolophus*	Forelimb reduction	HoxD genes (*GCR*) Differential growth rate
*Miniopterus*	Forelimb reduction	*Shh* Differential growth rate
*Dromaius*	Forelimb and sternal reduction and digit loss	*Tbx5*, *Msx2*, *Gli3*, *Shh* ^b^, *Grem1* ^b^, *Nkx2*.*5* Reduced growth rate
*Phalacrocorax*	Forelimb and sternal reduction	*Cux1* ^c^, *Ihh* ^b^ Reduced cartilage differentiation
*Gallus*	Hindlimb reduction	*Ihh* ^d^, *PTHrP* ^d^; or *C2CD3* ^d^ Attenuated proliferation; or loss of polarity

^a^ Gene modifications (top row) refer to expression changes unless otherwise noted. Resulting impact (bottom row) summarizes the suspected role of the gene modification(s) on development.

^b^ Altered expression thought to result from changes upstream.

^c^ Coding variant.

^d^ Unconfirmed mechanism.

## 2 TELEOST PELVIC FIN REDUCTION AND LOSS

Threespine sticklebacks (*Gasterosteus aculeatus*) are small teleost fish with populations in saltwater ocean and estuarine habitats, as well as freshwater lake and stream habitats (Bell & Foster, 1994; Schluter & McPhail, 1992). Marine threespine sticklebacks have robust bony armor that includes lateral plates, dorsal spines, and a pelvic girdle with spines. However, likely due to differences in water chemistry and predation regimes, freshwater sticklebacks usually evolve armor reduction, including reduction and/or loss of pelvic appendages (Bell et al., 1993; Giles, 1983; Hoogland et al., 1957; Reimchen, 1980, 1983, 1992, 2000; Smith et al., 2014; Spence et al., 2012, 2013; Zeller et al., 2012; Ziuganov & Zotin, 1995).

The stickleback pelvic appendage is a modified pelvic fin comprised of two articulated spines and a bony girdle that extends along the belly and up the sides of the fish. Over 100 geographically distinct freshwater populations of *G*. *aculeatus* have evolved reduction and/or loss of the pelvic spines and girdle (Bell et al., 1993; Chan et al., 2010; Coyle et al., 2007; Klepaker et al., 2013; Shapiro et al., 2006, 2009; Shikano et al., 2013). Because these freshwater populations were independently colonized by marine ancestors at the end of the last glacial maximum (Schluter & McPhail, 1992), they represent repeated instances of evolution and provide a good system for the study of genetic parallelism of appendage reduction and loss (Bolnick et al., 2018).

**FIGURE 3:**
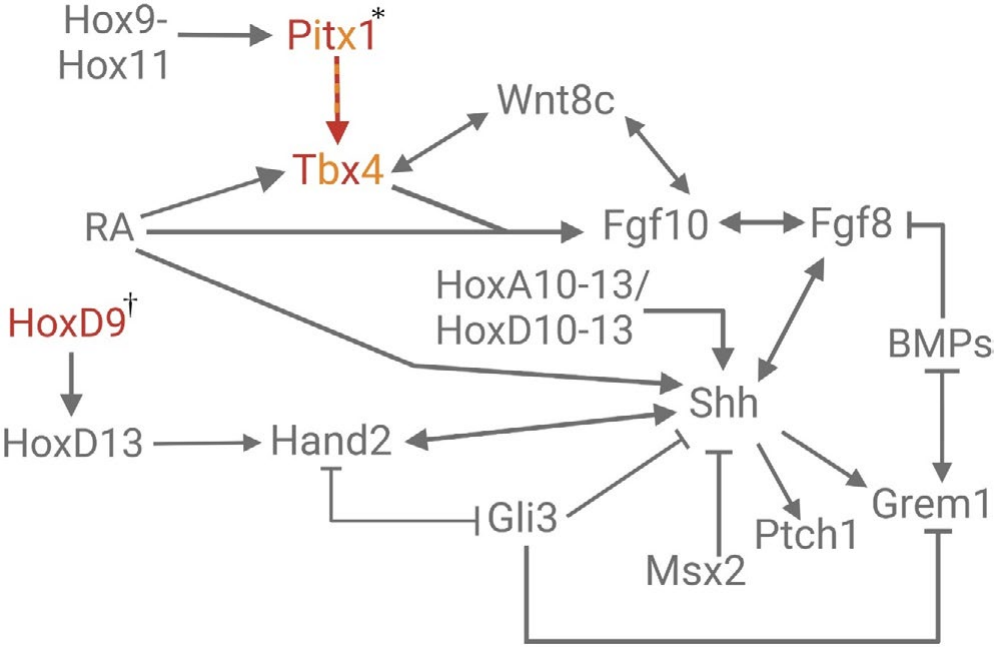
Gene regulatory network showing genes modified in stickleback* and fugu^†^. Gene symbols in red lettering mark the absence of expression in the appendage bud, while alternating red and orange gene names indicate that expression is either reduced or absent from the appendage bud depending on the individual. An orange arrow indicates decreased transactivation

Many instances of pelvic reduction in *G*. *aculeatus* have been linked to *Pitx1* (Bell et al., 2006; Coyle et al., 2007; Klepaker et al., 2013; Shapiro et al., 2006; Thompson et al., 2018). Relative to the pelvic‐complete morph, pelvic‐reduced *G*. *aculeatus* show no variation to their PITX1 amino acid sequences (Shapiro et al., 2006). Instead, pelvic‐complete and pelvic‐reduced morphs vary in *Pitx1* expression (Figure 3). *Pitx1* is expressed in the pelvis of pelvic‐complete larvae but is missing from the corresponding region of pelvic‐absent fish (Chan et al., 2010; Shapiro et al., 2006; Thompson et al., 2018). Reduction of *Pitx1* expression results in decreased transactivation of *Tbx4* (Figure 3), a gene important for appendage bud initiation and outgrowth (Cole et al., 2003; Don et al., 2016; Infante et al., 2013; Minguillon et al., 2005; Naiche & Papaioannou, 2007; Takeuchi et al., 2003; Tickle & Cole, 2004).

Pelvic expression of *Pitx1* in *G*. *aculeatus* is regulated by two pelvic‐specific enhancers—*PELA* and *PELB* (Chan et al., 2010; Thompson et al., 2018; Xie et al., 2019). Pelvic‐reduced sticklebacks have mutations in one or both enhancers and demonstrated reduced *Pitx1* expression in pelvic tissue (Chan et al., 2010; Kragesteen et al., 2018; Thompson et al., 2018; Xie et al., 2019). Genomic studies have shown that mutations to *PELA* arise de novo, likely because the enhancer is in a chromosomal region prone to double‐strand breakages (Xie et al., 2019). The *PELA* enhancer is subject to strong positive selection that drives the null allele to fixation (Chan et al., 2010; Xie et al., 2019). The strong selection for modified *Pitx1* regulation suggests a potential route to appendage reduction in other taxa if the lack of constraint is shared (Chan et al., 2010; Xie et al., 2019).

Indeed, more than thirty populations of the ninespine stickleback (*Pungitius pungitius*) have pelvic reduction and loss and show no differences in the PITX1 amino acid sequence between pelvic‐complete and pelvic‐absent fish (Klepaker et al., 2013; Shapiro et al., 2004, 2006). Instead, *Pitx1* expression is missing from the pelvic region of pelvic‐absent ninespines, as in threespine stickleback (Shapiro et al., 2004, 2006). Hybrids of threespine and ninespine stickleback with one pelvic‐complete parent and one pelvic‐reduced parent have a full pelvis, while hybrids with two pelvic‐reduced parents demonstrate pelvic spine and girdle reduction (Shapiro et al., 2006). These results indicate that pelvic reduction is controlled by regulation of the same locus, *Pitx1*, in threespine and ninespine sticklebacks, despite their 26‐million‐year divergence (Shapiro et al., 2006; Varadharajan et al., 2019). Moreover, modified *Pitx1* expression has been implicated in pelvic reduction of *G*. *doryssus*, a 10‐million‐year‐old threespine stickleback species from the Miocene (Stuart et al., 2020). This inference stemmed from an observation of pelvic asymmetry in which left side vestiges were larger than right side vestiges in *G*. *doryssus* fossils—a similar phenotype to that found in extant pelvic‐reduced stickleback (Nelson, 1971; Shapiro et al., 2004, 2006; Stuart et al., 2020). As such, it appears that pelvic reduction and loss in more than 100 populations across at least three stickleback species shares a genetic cause.

However, modified *Pitx1* expression does not drive pelvic appendage loss in a different teleost, the fugu (or pufferfish) *Takifugu rubripes*. Pelvic loss in fugu results instead from the absence of positional signaling by *HoxD9a* in the pelvic region. *HoxD9*, an orthologous gene, is important for appendage positioning and initiation in vertebrates (Figure 3) (Cohn et al., 1997; Tanaka et al., 2005). For example, in stickleback embryos, *HoxD9* expressed in pectoral and pelvic fin buds (Tanaka et al., 2005). In embryonic fugu, however, *HoxD9a* is expressed in the pectoral region but is absent from the pelvic region (Tanaka et al., 2005). Therefore, the absence of *Hoxd9a* expression in the pelvic region of fugu prevents fin and girdle formation.

## 3 SQUAMATE HINDLIMB REDUCTION AND LOSS

Squamate reptiles have independently evolved reduced limbs dozens of times (Brandley et al., 2008; Greer, 1991), most notably the snakes. No extant snake species retain forelimb or pectoral skeletal elements and most have no hindlimb or pelvic elements (Bellairs & Underwood, 1951; Cohn & Tickle, 1999; Vitt & Caldwell, 2013). However, basal snakes like the python (*Python regius*) have vestiges of the ilium and femur (Bellairs & Underwood, 1951; Cohn & Tickle, 1999; Hall, 2003; Leal & Cohn, 2016; Vitt & Caldwell, 2013).

**FIGURE 4:**
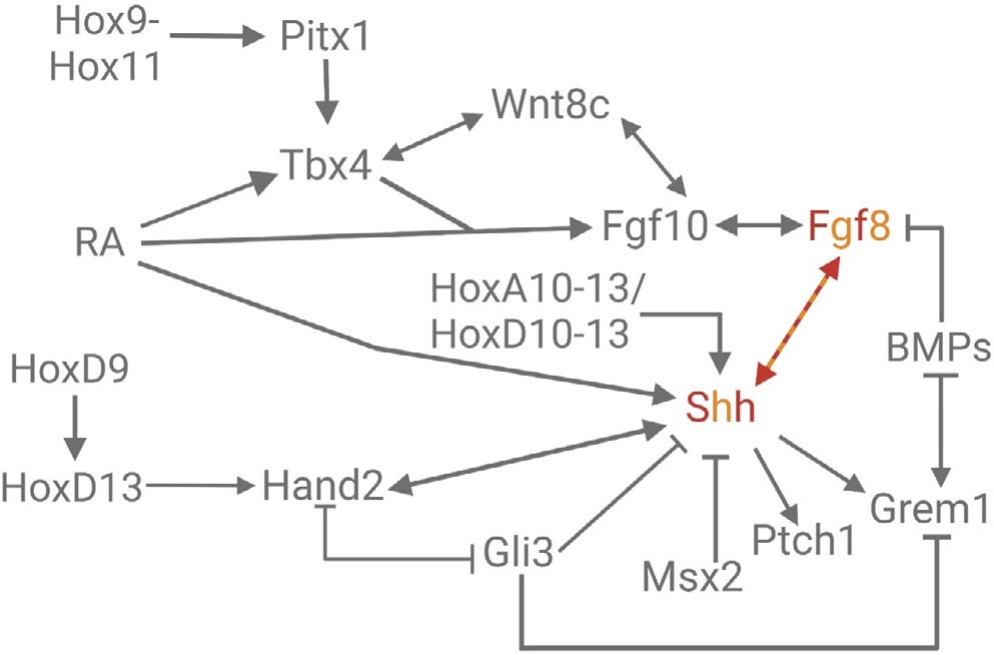
Gene regulatory network showing genes modified in *P*. *regius* hindlimb development. Gene symbols labeled in alternating red and orange letters indicate that expression is reduced and eventually terminates earlier than in typical tetrapod development. The alternating red and orange arrow indicates that the interaction between diminished Shh and Fgf8 results in arrested limb development

In typical tetrapods, SHH controls development along the anteroposterior axis of the limb bud, specifies bud width, and influences the presence and identity of digits (Chang et al., 1994; Chiang et al., 2001; Cohn & Tickle, 1999; López‐Martínez et al., 1995; Riddle et al., 1993; Ros et al., 2003). FGF8 is essential for distal growth of the limb bud (Boulet et al., 2004; Cohn & Tickle, 1999; Laufer et al., 1994; Neubüser et al., 1997; Ohuchi et al., 1997; Provot et al., 2008). Therefore, reciprocal regulatory interactions between SHH and FGF8 maintain gene expression and outgrowth in the developing limb (Figure 4) (Boulet et al., 2004; Cohn & Tickle, 1999; Leal & Cohn, 2016). In *P*. *regius*, development in hindlimb buds arrests early and then regresses because the feedback loop involving SHH and FGF8 is attenuated in the limb bud (Leal & Cohn, 2016).

*Shh* expression in the tetrapod limb is controlled by an enhancer called the *ZRS* (Galli et al., 2010; Lettice et al., 2003; Park et al., 2008; Riddle et al., 1993; Young & Tabin, 2017). The *P*. *regius ZRS* has three large deletion mutations relative to *Anolis sagrei*, a lizard with fully developed hindlimbs (Leal & Cohn, 2016). These mutations result in *Shh* expression that is reduced and terminates early (Leal & Cohn, 2016). Loss of SHH signaling is followed by a decrease in *Fgf8* expression, preventing limb and girdle growth in *P*. *regius* (Figure 4) (Leal & Cohn, 2016). Notably, *ZRS* sequences are even more poorly conserved in advanced snakes, likely driving complete loss of the hindlimb and pelvis (Kvon et al., 2016; Leal & Cohn, 2016).

## 4 SQUAMATE DIGIT LOSS

**FIGURE 5:**
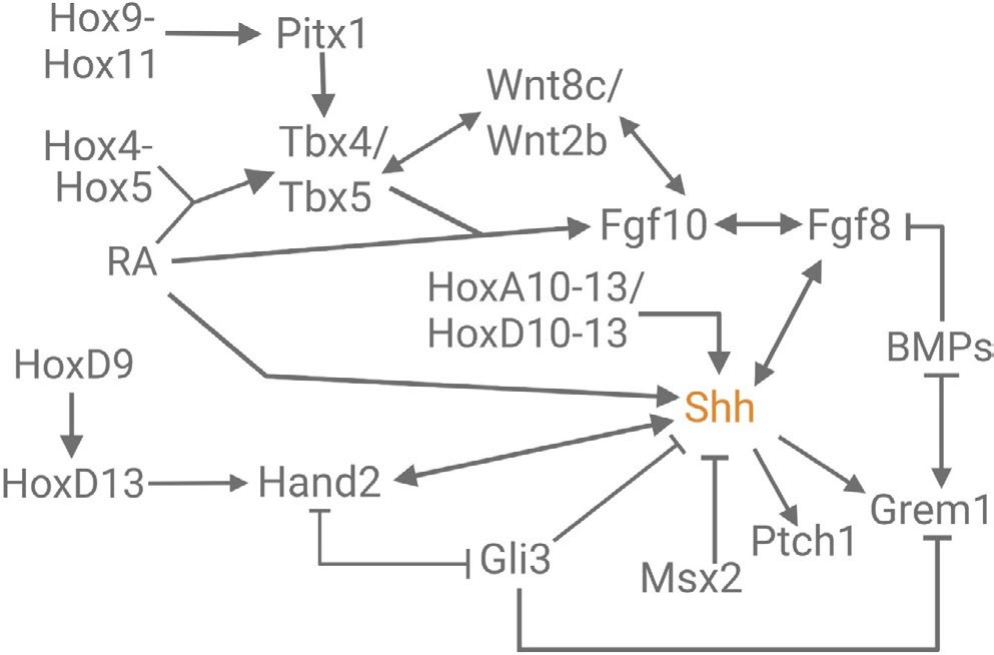
Gene regulatory network showing genes modified in digit loss of *Hemiergis* skinks. The duration of expression of *Shh*, shown in orange, varies between species

While less striking than the complete limb loss of snakes, digit loss in the fore‐ and hindlimbs of other nonsnake squamates has evolved over twenty separate times (Brandley et al., 2008; Greer, 1991). Scincidae, a squamate family with over 1700 described species, accounts for nearly half of these instances of digit loss (Brandley et al., 2008; Uetz et al., n.d.). For example, fore and hindlimb digit number varies between the seven species of the Australian genus *Hemiergis* (Shapiro et al., 2003; Uetz et al., n.d.). *Hemiergis initialis* retains five digits on each limb, whereas *H*. *peronii* has lost 2 digits on every limb and *H*. *quadrilineata* has lost three digits on every limb (Shapiro et al., 2003). Variation in *Hemiergis* digit number correlates with the duration of *Shh* expression in the limb bud: shorter expression corresponds to fewer digits (Figure 5) (Shapiro et al., 2003).

## 5 MAMMAL HINDLIMB LOSS AND PELVIC GIRDLE REDUCTION

**FIGURE 6:**
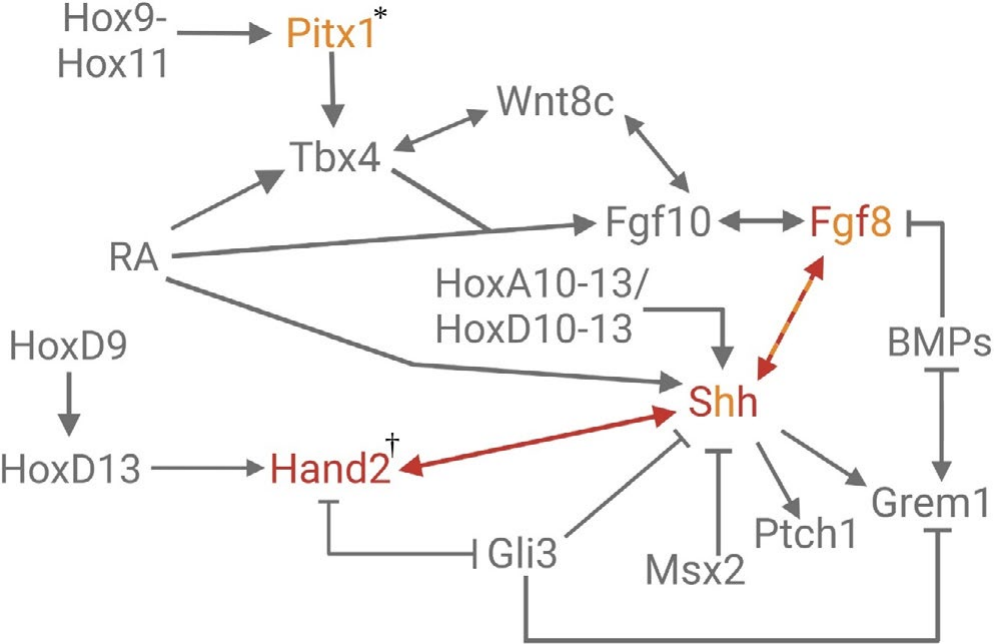
The gene regulatory network modified in the reduction of hindlimb and pelvic elements in cetaceans^†^ and sirenians*. Gene symbols in red are not expressed in the hindlimb bud. Alternating red and orange lettering indicates that gene expression is reduced and terminates earlier than in typical limb development. The alternating red and orange arrow indicates that the interaction between diminished *Shh* and *Fgf8* results in arrested limb development. Modified expression of *Pitx1*, written in orange, is suspected to underlie hindlimb loss and pelvic reduction in manatee

Sirenians (manatees and dugongs) and cetaceans (dolphins, porpoises, and whales) are aquatic mammal lineages that have independently evolved hindlimb loss and pelvic reduction (Adam, 2009; Senter & Moch, 2015; Springer et al., 2004; Thewissen et al., 2001, 2006). In the spotted dolphin (*Stenella attenuatus*), HAND2, an activator of *Shh*, is absent from the embryonic hindlimb bud (Charité et al., 2000; Fernandez‐Teran et al., 2000; Galli et al., 2010; Ros et al., 2003; Thewissen et al., 2006). This prevents *Shh* initiation which in turn diminishes *Fgf8* expression (Figure 6) (Ros et al., 2003; Thewissen et al., 2006). As noted in *P*. *regius*, FGF8 is initially present in the cetacean hindlimb bud but is not sustained without *Shh* expression (Richardson & Oelschläger, 2002; Sedmera et al., 1997; Thewissen et al., 2006; Zhu et al., 2008). This results in the attenuation of limb outgrowth, regression of the limb bud, and reduction to a vestigial pelvis (Bejder & Hall, 2002; Cooper, 2009; Sedmera et al., 1997; Thewissen et al., 2006; Zhu et al., 2008).

The molecular origins of sirenian loss and reduction have yet to be explored, but their pelvic morphology offers some insight. In mice, humans, and stickleback, reduction in PITX1 level or function results in pelvic appendage vestiges that are, on average, larger on the left side than the right (Alvarado et al., 2011; Chan et al., 2010; Gurnett et al., 2008; Kragesteen et al., 2018; Lanctôt et al., 1999; Marcil et al., 2003; Shapiro et al., 2004, 2006; Shiratori et al., 2014; Szeto et al., 1999; Thompson et al., 2018). Reduction or loss of *Pitx1* in the posterior appendage unmasks the asymmetrical expression of *Pitx2*, one of only six genes known to generate left‐larger directional asymmetry in limb bud (Palmer, 2004). Pelvic vestiges of the manatee (*Trichechus manatus latirostris*) demonstrated this characteristic asymmetry: out of 114 skeletal specimens, 93 had larger left side pelvic vestiges, thus implicating *Pitx1* in hindlimb loss and pelvic reduction in manatee (Figure 6) (Shapiro et al., 2006).

## 6 MAMMAL DIGIT REDUCTION

The number and size of digits is variable among mammals; more than half of mammalian orders demonstrate some form of digit reduction (Sears et al., 2011). The first digit in all adult even‐toed ungulates (order Artiodactyla) is absent, and digits II and V are reduced in length or lost in many species (Lopez‐Rios et al., 2014; Sears et al., 2011). For example, in the pig (*Sus scrofas*), digits II and V are reduced to vestigial dewclaws while in cow (*Bos taurus*) and camel (*Camelus dromedarius*) these digits are absent (Cooper et al., 2014; Lopez‐Rios et al., 2014; Sears et al., 2006; Tissières et al., 2020). Digit reduction and loss in pig and cattle develop similarly. In both pig and cow, expression of *Ptch1*, an important SHH signal transducer, is restricted and symmetrical relative to the pentadactyl limb (Lopez‐Rios et al., 2014; Tissières et al., 2020). In cow, two insertions in an enhancer called the *LRM* drive restricted *Ptch1* expression (Lopez‐Rios et al., 2014); similar modifications might restrict *Ptch1* expression in pigs. As a result, SHH targets like *Gli1*, *Grem1*, and HoxD genes are expressed in a more symmetrical pattern compared to the mouse limb (Cooper et al., 2014; Lopez‐Rios et al., 2014; Tissières et al., 2020). Following the loss of asymmetry, *Fgf8* expression is reduced at the distal tip of digits II and V and results in the reduced length of the dewclaws in pigs (Cooper et al., 2014; Lopez‐Rios et al., 2014; Tissières et al., 2020). Similarly, *Fgf8* expression is absent from digits II and V in cow, leading to digit loss (Cooper et al., 2014; Lopez‐Rios et al., 2014; Tissières et al., 2020).

Unlike pig and cow, loss of digits II and V in camels proceeds by apoptosis in the digit forming regions of the limb bud (Cooper et al., 2014; Lopez‐Rios et al., 2014; Sears et al., 2011). *Msx2* and *Bmp4*, markers of apoptosis, are upregulated in digits II and V (Cooper et al., 2014). Accordingly, the rate of cell death is elevated relative to cow and pig (Cooper et al., 2014). Therefore, camel digit reduction proceeds via sculpting of the limb bud by cell death. (Cooper et al., 2014; Lopez‐Rios et al., 2014; Sears et al., 2011). A similar apoptotic mechanism is involved in the loss of digits I and V in the hindlimb of the three‐toed jerboa (*Dipus sagitta*) and digits II and IV of horse (*Equus ferus caballus*) (Cooper et al., 2014; Zúñiga, 2015).

## 7 MAMMAL FORELIMB REDUCTION

The order Chiroptera contains over 1400 species of bats, the only mammals capable of powered flight (Lei & Dong, 2016; “Mammal Diversity Database (Version 1.5),” 2021; Simmons et al., 2008). Flight evolved early in the bat lineage and was facilitated by substantial changes to forelimb and pectoral girdle structure, including reduction in bone size (Hockman et al., 2008; Simmons et al., 2008). Specifically, the length and width of the ulna are reduced relative to the radius, with the distal tip of the ulna fused to the radius (Sears, 2008; Sears et al., 2007). Ulnar reduction decreases wing weight without compromising its function (Sears, 2008).

Ulnar width reduction in bats results from differential growth rates between the radius and the ulna (Sears et al., 2007). In the short‐tailed fruit bat (*Carollia perspicillata*) and the little brown bat (*Myotis lucifugus*), the cartilage condensations that will form the radius and ulna are initially similar in width (Adams, 1992; Sears et al., 2007). The relative width of the ulna begins to decrease with the onset of ossification, and it continues to narrow as the distal tip fuses to the radius (Adams, 1992; Sears et al., 2007). Two processes have been suggested to cause ulnar width reduction: (a) abnormal morphology of differentiating cartilage cells or (b) a lower rate of bone deposition (appositional growth) (Biga et al., n.d.; Sears et al., 2007).

Ulnar length reduction likely results from modified regulation of essential limb patterning genes. In *C*. *perspicillata*, *M*. *lucifugus*, and *Miniopterus schreibersii* (the common bent‐wing bat), posterior HoxD gene expression is upregulated and prolonged in the developing wing relative to the hindlimb or mouse limbs (Chen et al., 2005; Ray & Capecchi, 2008; Wang et al., 2014). Additionally, the anterior edge of *HoxD13* expression is shifted distally, and the posterior edge is shifted proximally in the bat forelimb bud (Chen et al., 2005; Ray & Capecchi, 2008). HoxD *cis*‐regulatory elements have bat‐specific changes that are not shared with other mammals (Booker et al., 2016; Ray & Capecchi, 2008). For example, the *GCR* is a regulatory region that drives HoxD gene expression in the mammalian forelimb (Ray & Capecchi, 2008). Compared to mouse or human GCRs, the Chiropteran *GCR* has several lineage‐specific sequences and drives altered expression of HoxD genes when compared to mouse or human *GCR*s (Ray & Capecchi, 2008). Altered expression of HoxD genes results in aberrations in ulnar length (Boulet & Capecchi, 2004; Chen et al., 2005; Hérault et al., 1997; Peichel et al., 1997; Ray & Capecchi, 2008; Sears, 2008).

In the Natal long‐fingered bat (*Miniopterus natalensis*), *Shh* expression is delayed but spatially expanded in the forelimb bud, relative to mouse (Hockman et al., 2008). In experimental studies, *Shh*‐knockout mice showed reduced cell proliferation and increased cell death in forelimb buds, resulting in a mutant phenotype similar to the batwing—a normal radius and a reduced ulna (Ahn & Joyner, 2004; Chiang et al., 2001; Hockman et al., 2008; Sears, 2008). This change in *Shh* expression might also contribute to the expanded Hox gene expression that shrinks the ulna, discussed above (Chiang et al., 2001; Hockman et al., 2008).

## 8 BIRD FORELIMB AND STERNAL REDUCTION

The emu, *Dromaius novaehollandiae*, is a flightless bird with reduced sternum, humerus, radius, ulna, and autopodial elements (Bickley & Logan, 2014; Farlie et al., 2017; Kawahata et al., 2019; Maxwell & Larsson, 2007; Smith et al., 2016; Vokes et al., 2008). Wing morphology is highly variable between and even within individuals. Digit III is the only digit retained across individuals, though vestigial digits II and/or IV are commonly fused to digit III (Farlie et al., 2017; Kawahata et al., 2019; Maxwell & Larsson, 2007; Vokes et al., 2008). The variable forelimb reduction and digit loss suggests that emu wing morphology is not constrained (Farlie et al., 2017; Kawahata et al., 2019; Maxwell & Larsson, 2007; Vokes et al., 2008).

Expression of *Tbx5* in the emu wing bud is delayed relative to chick, reducing recruitment of progenitor cells in sternal and forelimb tissues (Bickley & Logan, 2014; Minguillon et al., 2005; contra Farlie et al., 2017). With fewer progenitor cells, rates of proliferation and outgrowth are reduced, and the emu wings grow 64% slower than chicken wings (Bickley & Logan, 2014; Farlie et al., 2017; Faux & Field, 2017; Smith et al., 2016). Notably, the emu wing bud emerges after and develops more slowly, than the hindlimb bud (Ahn & Joyner, 2004; Bickley & Logan, 2014; Butterfield et al., 2009).

**FIGURE 7:**
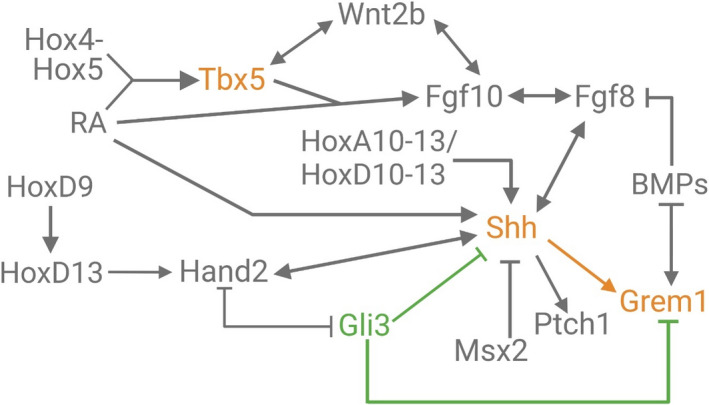
Gene regulatory network modified in the reduction in the emu forelimb and sternum. Expression of genes in orange is reduced while genes in green are upregulated. *O*range arrows demonstrate decreased activation of *Grem1* by Shh while increased inhibitory activity is shown in green

S*hh* expression in the wing is also delayed and decreased relative to the emu hindlimb and the chick wing. Two *Shh* repressors, *Msx2* and *Gli3*, are upregulated in the emu forelimb relative to its hindlimb (Figure 7) (Bakker et al., 2013; Smith et al., 2016). Experimental expression of *Msx2* in the chick wing bud led to a reduction in the number and length of wing elements and produced an emu‐like wing (Ferrari et al., 1998; Smith et al., 2016; Welscher et al., 2002; te Welscher, Zuniga, et al., 2002). *Gli3* is important for regulating digit number, so overexpression could result in digit loss (Litingtung et al., 2002; Lopez‐Rios et al., 2012; Paese et al., 2021; te Welscher, Zuniga, et al., 2002; Zúñiga & Zeller, 1999). *Grem1*, another gene important for digit patterning, is repressed by GLI3 but upregulated and maintained by SHH and HAND2 (Kawahata et al., 2019; Litingtung et al., 2002; Panman & Zeller, 2003; Vokes et al., 2008; te Welscher, Fernandez‐Teran, et al., 2002; te Welscher, Zuniga, et al., 2002; Zúñiga et al., 1999) (Figure 7). Restriction of *Shh* expression and upregulation of *Gli3* in the emu forelimb reduces *Grem1* expression relative to chick, thereby decreasing digit number (Figure 7) (Farlie et al., 2017; Kawahata et al., 2019; Lopez‐Rios et al., 2012; Smith et al., 2016; Vokes et al., 2008).

*Nkx2.5* is expressed in the forelimb of early emu embryos but not in the chicken, zebra finch, or ostrich wing buds which develop into typical three‐digit wings (Farlie et al., 2017). Experimental expression of *Nkx2.5* in chick wing buds resulted in reduced distal wing elements and emu‐like wings (Farlie et al., 2017). *Nkx2.5* might also influence forelimb reduction in the kiwi and cassowary (Farlie et al., 2017), two wing‐reduced species closely related to the emu (Farlie et al., 2017; Faux & Field, 2017; Harshman et al., 2008; Mitchell et al., 2014; Phillips et al., 2009; Sackton et al., 2019).

The flightless Galápagos cormorant (*Phalacrocorax harrisi*) has a short radius and ulna relative to its humerus (Bickley & Logan, 2014; Burga et al., 2017). Compared to flying cormorant species, the Galápagos cormorant has a deletion of four amino acids in the CUX1 coding sequence (Burga et al., 2017). In experiments with mouse cell lines, the resultant protein was less effective in activating *Ihh*, a gene important for the proliferation and differentiation of cartilage cells (Burga et al., 2017; Kronenberg, 2003; Peckham et al., 2003).

## 9 BIRD HINDLIMB REDUCTION

In all extant birds and their recent ancestors, the fibula is splinter‐like and reduced, usually around 2/3 length of the tibia (Botelho et al., 2016; Paese et al., 2021). Initially, the two cartilaginous elements that form the tibia and fibula are approximately equal in size (Botelho et al., 2016; Paese et al., 2021). In one possible explanation, the fibula is reduced because it lacks a distal growth plate (Botelho et al., 2016). Without the growth plate, the fibula does not maintain a population of immature, proliferating cartilage cells that drive distal growth because the feedback loop between IHH and PTHrP is disrupted. Indian Hedgehog encourages the formation of bone from cartilage and the production of PTHrP (Botelho et al., 2016). Conversely, PTHrP delays cartilage maturation and inhibits IHH production (Botelho et al., 2016). The distal portion of the fibula does not maintain PTHrP expression, but the fibulare acts as a surrogate growth plate early in bone development. While the fibulare is appressed to the fibula, it provides PTHrP signaling that inhibits IHH production and allows for continued cartilage growth (Botelho et al., 2016). Over the course of bone development, the fibulare separates from the fibula and PTHrP signaling no longer reaches distal cartilage of the fibula (Botelho et al., 2016). Without PTHrP to maintain the feedback loop with IHH, the growth of the fibula is slow and terminates early, resulting in a short, splinter‐like bone (Botelho et al., 2016).

Another explanation is that altered Hedgehog signaling disrupts anteroposterior polarity in the developing bird hindlimb (Paese et al., 2021). The *talpid^2^* mutant chicks, a 19‐bp deletion in *C2cd3* prevents formation of the repressive form of GLI3 (Paese et al., 2021). This mutation leads to ectopic SHH signaling, polydactyly, degradation of digit identity and autopod asymmetry, and fibular extension (Paese et al., 2021). That is, in *talpid^2^* chicks, the lengths of the tibia and fibula remain similar throughout development, while the tibia extends significantly relative to the wild‐type fibula (Botelho et al., 2016; Paese et al., 2021). Thus, evolutionary changes in the regulation of Hedgehog signaling might drive development of the reduced fibula in normal birds. This model could also explain digit loss in the bird hindlimb (Litingtung et al., 2002; Lopez‐Rios et al., 2012; Paese et al., 2021; te Welscher, Zuniga, et al., 2002; Zúñiga & Zeller, 1999).

## 10 CONCLUSION

Convergence on appendage reduction and loss across vertebrates suggests that natural selection has repeatedly favored this phenotype. We found that appendage reduction and loss are underlain by a mix of shared and unique molecular mechanisms, depending on taxon and limb position (Table 2). Sears et al. (2007) noted a similar pattern in mammalian zeugopod reduction: while the timing and mechanism of fibula width reduction is shared between the bat *C*. *perspicillata* and mouse, the mechanisms underlying additional convergent morphological characteristics vary. For another example, *Pitx1* expression is repeatedly modified within and among stickleback species, suggesting parallel evolution within that lineage. On the other hand, altered regulation of *Pitx1* does not influence pelvic reduction in fugu, nor in any of the other vertebrate groups surveyed here (except possibly manatee).

The mechanism most often shared among taxa was modulation of *Shh* expression and signal transduction, which was associated with limb reduction in squamates, cetaceans, artiodactylans, bat, and emu. The central role of SHH in limb patterning and outgrowth likely influences in its parallel modification in distantly related vertebrate clades. However, the specific molecular mechanisms by which SHH levels were altered vary by taxon and limb type. For example, *cis*‐regulatory mutations attenuated *Shh* expression in the python hindlimb while altered activator (*Hand2*) and repressor (*Msx2* and *Gli3*) expression reduced *Shh* in the cetacean hindlimb and emu forelimb, respectively. Hox genes were similarly implicated in multiple instances of appendage reduction or loss, though again modifications differed between taxon and appendage type. For example, fugu pelvic fin loss resulted from a lack of *HoxD9a* expression in the pelvic region while altered HoxD *cis*‐regulation likely drove bat ulnar reduction.

Perhaps it is unsurprising that vertebrate appendage reduction and loss is underlain by both shared and unique molecular mechanisms. Appendage development is controlled by spatially and temporally regulated expression of dozens of interacting genes—a complexity that creates potential for numerous routes to appendage reduction and loss. However, many key developmental genes have pleiotropic effects across the body plan, so evolution could be constrained to only a handful of pathways. Such constraint may explain the most salient finding of our review: in all cases but one, appendage reduction and loss resulted not from changes in protein coding DNA but from changes to enhancer sequences and limb‐specific gene expression patterns. The evolutionary importance of regulatory mutations is contentious, especially for gain‐of‐function adaptations (Hoekstra & Coyne, 2007). However, our findings support the assertion that regulatory changes represent a major mode of evolution because of their repeated role in loss‐of‐function phenotypes that are likely adaptive (Chan et al., 2010; Hoekstra & Coyne, 2007).

## CONFLICT OF INTEREST

Authors involved in preparation of this manuscript have no conflicts of interest to declare.

## AUTHOR CONTRIBUTION

**Samantha Swank:** Conceptualization (equal); Investigation (lead); Methodology (equal); Project administration (lead); Visualization (lead); Writing‐original draft (lead); Writing‐review & editing (equal). **Thomas J. Sanger:** Validation (equal); Writing‐review & editing (supporting). **Yoel E. Stuart:** Conceptualization (equal); Funding acquisition (lead); Methodology (equal); Validation (equal); Writing‐review & editing (equal).

## Data Availability

No datasets were generated or analyzed in production of this review.
